# Evaluation and Management of Transitional Fractures of the Distal Radius

**DOI:** 10.7759/cureus.78353

**Published:** 2025-02-01

**Authors:** Tyler J Moon, Julian A Gatta, James R Anderson, Jochen P Son-Hing

**Affiliations:** 1 Department of Orthopedic Surgery, University Hospitals Cleveland Medical Center, Cleveland, USA; 2 Department of Orthopedic Surgery, University Hospitals Rainbow Babies and Children's Hospital, Cleveland, USA

**Keywords:** closed reduction, distal radius fractures, growth arrest, physeal fractures, transitional fractures

## Abstract

Introduction: Transitional fractures about a closing physeal plate in adolescents are most common at the distal tibia physis but have been reported in other anatomical locations, including the distal radius. Only 10 prior cases of transitional fractures of the distal radius have been reported in the prior literature. This study aims to report the findings and outcomes from the largest series to date of these rare injuries.

Methods: Data were collected on six cases of these fractures in patients less than 18 years of age. Demographic, injury, radiographic, and outcome data were collected via retrospective chart review.

Results: The patients' ages ranged from 13 to 18 years. Four patients were male and two were female. Four of six patients presented after low-energy falls during sporting events. None were open injuries, and there was no or minimal displacement in all cases. The typical radiographic pattern in both this series and prior reports was an ulnar-sided Salter-Harris (SH) III fracture on the anterior-posterior (AP) radiograph and an SH-II fracture on the lateral radiograph. Two injuries had an isolated SH-III component on the AP radiograph, which had only been reported once prior. Five patients were treated with closed reduction and casting. One patient was treated with closed reduction and percutaneous pinning (CRPP) due to loss of reduction after splinting and involvement of the distal radio-ulnar joint (DRUJ). To the best of our knowledge, this is the first reported case to be managed in this fashion. All injuries had routine healing without complications, other than one case that developed partial growth arrest and required physeal bar excision with < 1 cm limb length discrepancy and no functional deficit at 18 months after physeal bar excision.

Conclusions: This is the largest series of transitional fractures of the distal radial physis presented to date and broadens the resources available for the evaluation and management of these rare injuries. This series additionally demonstrates fracture patterns similar to the Tillaux fracture of the distal tibia, with an isolated SH-III fracture component in the coronal plane, and demonstrates successful treatment with CRPP, both of which can help guide diagnosis and treatment in the future.

## Introduction

Epiphyseal injuries of a partially closed growth plate in adolescents, also known as transitional fractures, present unique challenges in management. Long-term sequelae, such as early degeneration and chronic pain, are a concern without appropriate articular reduction, as is the potential for growth arrest [1]. These injuries most commonly occur about the distal tibial physis but have also been described at the distal humerus [2], distal femur [3], proximal tibia [4], foot [5], and distal radius [6-13]. Although distal radius fractures are extremely common in the pediatric population, transitional fractures of the distal radial physis are rare, likely due to the rapid rate of physeal closure at this location [14]. Only 10 cases of transitional distal radius fractures in adolescents have been reported in the prior literature [6-13]. The following series of six transitional distal radius fractures is the largest presented to date and aims to expand on prior knowledge regarding diagnosis, treatment options, and possible complications from this uncommon injury.

## Materials and methods

Cases of transitional distal radius fractures in adolescent patients were collected at University Hospitals Rainbow Babies & Children's Hospital, University Hospitals Cleveland Medical Center, a single level 1 pediatric trauma center in Cleveland, OH, after informed consent. Cases were identified based on a retrospective review of adolescent distal radius fractures from January 1, 2005, through December 31, 2020. Patients were included if they sustained a physeal distal radius fracture through a partially ossified physis or identified as any percentage of incomplete ossification on plain radiographs. Patients were excluded if they sustained a fracture through a non-ossified (0% ossification) or completely ossified (100% ossification) physis.

The following parameters were collected retrospectively: age at injury, mechanism of injury, time to presentation, reported symptoms, open fracture, neurovascular injury, concomitant injuries, radiographic characteristics of injury, management methods, duration of immobilization, outcome, and complications including growth arrest. All radiographic assessments were completed by the authors and were corroborated with the initial radiology attending read. Management methods were collected via clinic and operative notes when applicable. Patients were followed up until clinical and radiographic healing at the time of discharge from follow-up as per standard protocol at the study institution, defined as full asymptomatic function and absence of fracture line on plain radiographs. Patients were followed up for the duration of care if complications developed. All data were stored electronically. As this was a descriptive study, no statistical analysis was completed. 

## Results

This series includes six cases of transitional fractures of the distal radius in six different adolescent patients (Table 1). The age range of patients within the series was 13 to 18 years. Four patients were male and two were female. Mechanisms of injury included four falls (three of which occurred during sporting events), one all-terrain vehicle (ATV) accident, and one pedestrian struck by a vehicle. Four patients presented to the emergency department immediately after the injury, while two presented to the outpatient clinic 24-48 hours after an initial primary care or urgent care visit. The only symptoms in each of the six cases were swelling and tenderness at the distal forearm and pain leading to limitations of wrist range of motion. None of the injuries were open, and none had associated neurovascular injury. Five of the injuries were isolated, while one patient (case II) had an ipsilateral right clavicle fracture and a contralateral left posterior cruciate ligament avulsion.

**Table 1:** Patient characteristics and management M: male; F: female; ATV: all-terrain vehicle; CRPP: closed reduction and percutaneous pinning

Case	Age	Sex	Mechanism	Displaced?	Is closed reduction required?	Treatment method	Outcome
I	16	M	Fall from a tree	< 2 mm	No	Short arm casting	Healed and asymptomatic at six weeks
II	13	M	ATV rollover	< 2 mm	Yes	Short arm casting	Growth arrest and physeal bar excision, no functional limitations at 18 months post-excision
III	18	M	Fall, sports	No	No	Short arm casting	Healed and asymptomatic at six weeks
IV	15	M	Fall, sports	< 2 mm	Yes	Short arm casting	Healed and asymptomatic at six weeks
V	15	F	Fall, sports	> 2 mm	Yes	Failed closed reduction -> CRPP	Healed and asymptomatic at six weeks after pin removal
VI	14	F	Pedestrian struck	< 2 mm	No	Short arm casting	Healed and asymptomatic at six weeks

**Figure 1:**
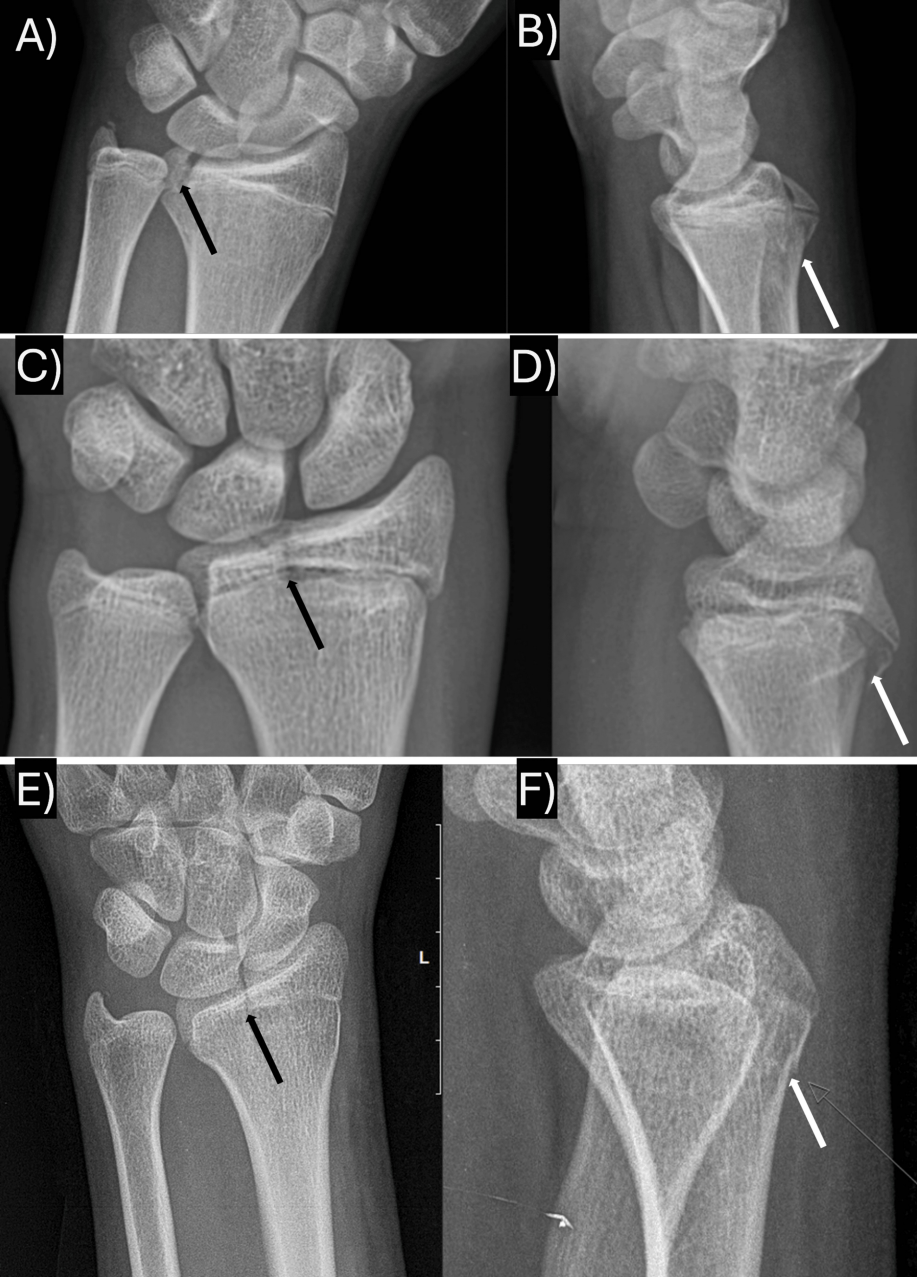
Injury films of cases with SH-III components on AP radiographs and SH-II components on lateral radiographs A) Case I's AP radiographs; B) Case I's lateral radiographs; C) Case IV's AP radiographs; D) Case IV's lateral radiographs; E) Case VI's AP radiographs; F) Case VI's lateral radiographs. In all panels, the black arrows demonstrate the AP radiograph SH-III component and the white arrows demonstrate the lateral radiograph SH-II component. SH: Salter-Harris; AP: anterior-posterior

**Figure 2:**
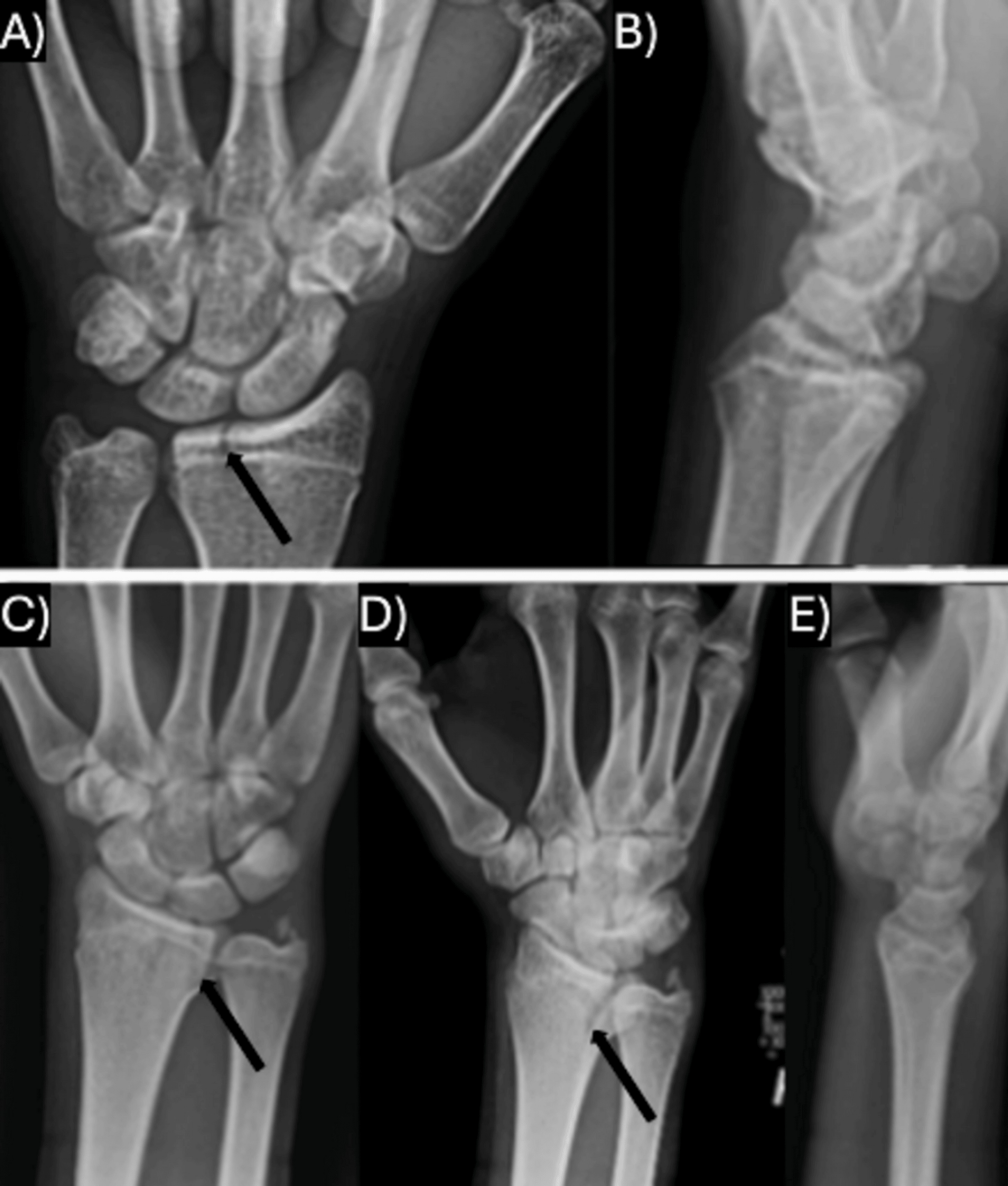
Injury films of cases with isolated SH-III components on AP radiographs and no fracture lines on lateral radiographs A) Case III's AP radiographs; B) Case III's lateral radiographs demonstrating no fracture line. C) Case V's AP radiographs; D) Case V's oblique radiographs; E) Case V's lateral radiographs demonstrating no fracture line. For Figures A, C, and D, the black arrow indicates the isolated SH-III component on the AP/oblique radiographs. SH: Salter-Harris; AP: anterior-posterior

**Figure 3:**
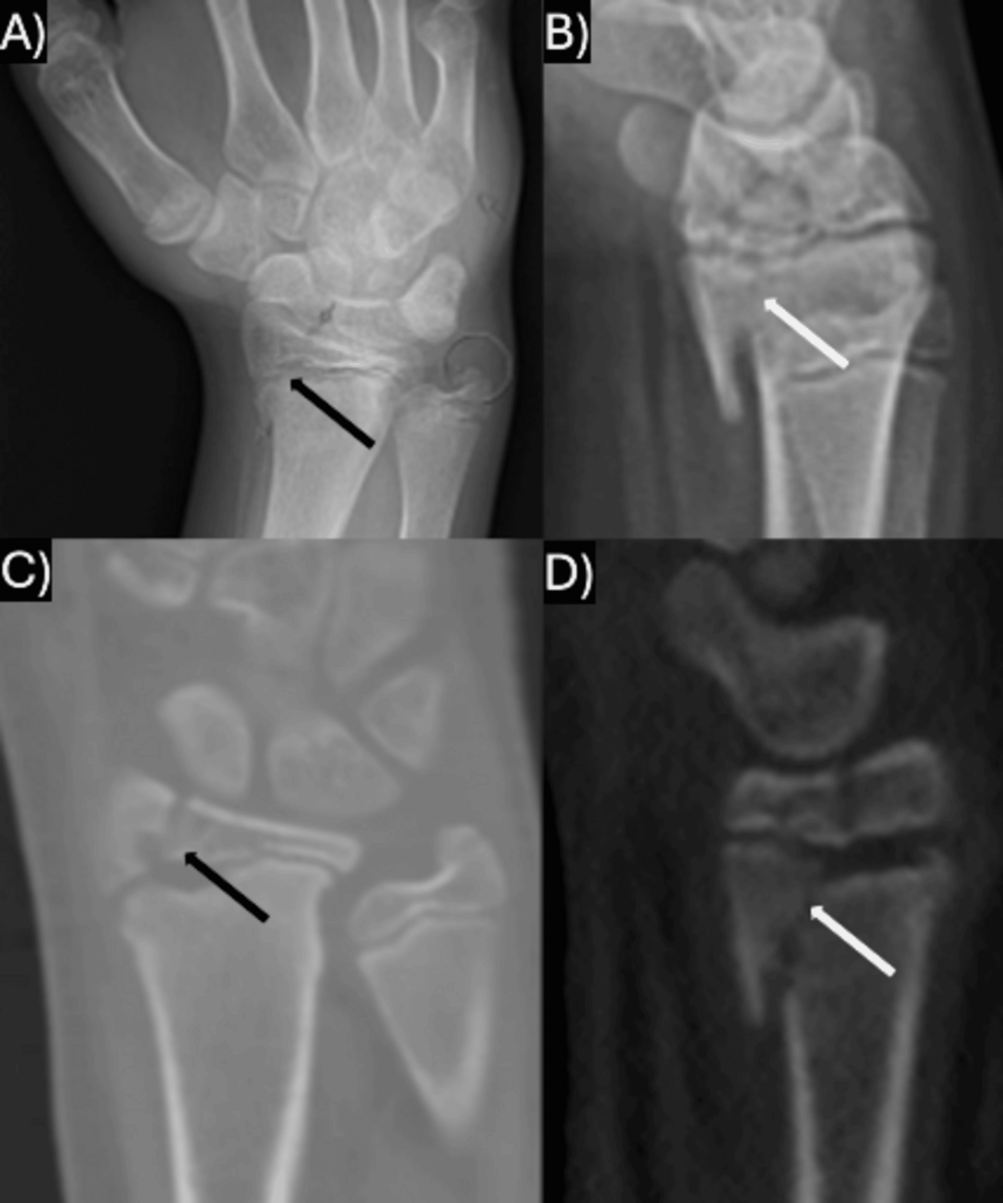
Imaging from case II: this was the only injury with a radial-based fracture as well as an SH-IV fracture line on the lateral radiographs. A) Case II's AP radiographs; B) Case II's lateral radiographs; C) Case II's coronal CT imaging; D) Case II's sagittal CT imaging. In all panels, the black arrows demonstrate the AP radiograph/coronal CT fracture line, while the white arrows demonstrate the lateral radiograph/sagittal CT fracture line. SH: Salter-Harris; AP: anterior-posterior

In three cases (cases I, IV, and VI), plain radiography detected the complex physeal fracture, each with an ulnar-sided Salter-Harris (SH) III component on the anterior-posterior (AP) radiograph and a dorsal SH-II component on the lateral radiograph (Figure 1). One of these patients had a concomitant ulnar styloid fracture, and each of these injuries was minimally displaced. Two cases (case III and V) were found to have only an isolated ulnar-sided SH-III fracture on the AP radiograph (Figure 2). Case V had an ulnar styloid fracture and involvement of the distal radio-ulnar joint (DRUJ). Case II was found to have a radial-sided SH-III component on the AP radiograph and a volar SH-IV component on the lateral radiograph with mild translation (Figure 3). This fracture pattern was only detected on a computed tomography (CT) scan (it was thought to be only a volar SH-II fracture on plain radiography), which was ordered due to the high-energy mechanism of the injury and because the patient required a CT as part of a trauma workup (Table 2).

**Table 2:** Radiographic characterization of fractures SH: Salter-Harris; DRUJ: distal radio-ulnar joint; N/A: not available

Case	Radial- or ulnar-sided	Pattern on AP radiograph	Pattern on the lateral radiograph	Ulnar styloid fracture	Other features
I	Ulnar	SH-II	SH-III	Yes	N/A
II	Radial	SH-IV	SH-III	Yes	Moderate comminution, volar translation
III	Ulnar	N/A	SH-III	No	N/A
IV	Ulnar	SH-II	SH-III	No	Dorsal translation
V	Ulnar	N/A	SH-III	Yes	Dorsal translation, involvement of DRUJ
VI	Ulnar	SH-II	SH-III	No	N/A

All patients were acutely managed in a sugar tong splint placed either in the emergency room or the orthopedic clinic. In the one case that was non-displaced, the fracture was splinted in situ. In the other five cases that were displaced, closed reduction in the emergency room was achieved via traction, a recreation of the deformity, and direct pressure over the displaced fragment. All fractures were molded with the wrist in neutral and using a three-point mold to counteract the potential displacement of the distal fragment.

**Figure 4:**
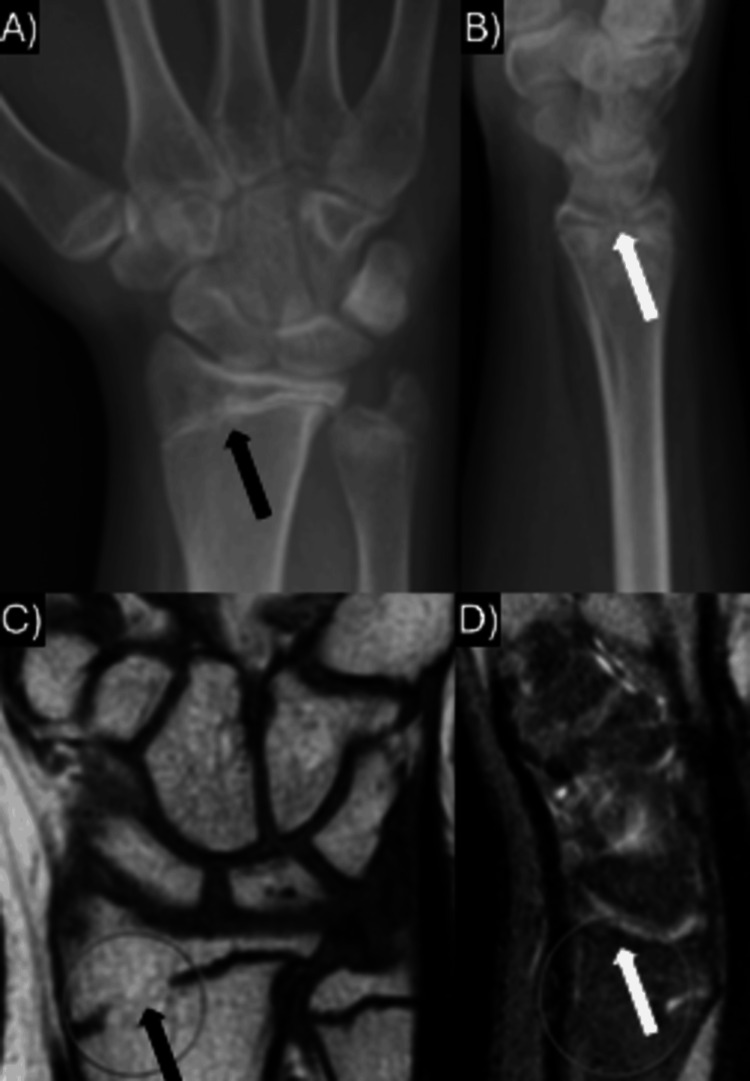
Plain radiographs and MRI of case 11 obtained at the 15-month follow-up showing decreased radial inclination and radially-based physeal bar formation. A) Case II's AP plain radiographs at the 15-month follow-up; B) Case II's lateral plain radiographs at the 15-month follow-up; C) Case II's coronal T1 MRI imaging at the 15-month follow-up; D) Case II's sagittal T1 MRI imaging at the 15-month follow-up. Black arrows in panes A and C demonstrate the physeal bar on AP radiographs and coronal MRI imaging, while white arrows in panes B and D demonstrate the physeal bar on lateral radiographs and sagittal MRI imaging. AP: anterior-posterior

Four patients had repeat imaging between seven and 10 days, which showed maintenance of fracture alignment (cases I, III, IV, and VI). Case II underwent repeat imaging out of the splint in the operating room during the management of his other injuries; these images confirmed maintenance of reduction, and the patient was placed back into a sugar-tong splint. These patients were each placed in a short arm cast, as is standard at the study institution for distal radius fractures after the initial swelling subsided and were immobilized for a total of five to six weeks. At the time of cast removal, each of the five patients had limited wrist range of motion but no pain or tenderness at the wrist. They were all withheld from contact sports with protected weight bearing for one week after removal but were allowed to begin range of motion as tolerated, as is standard protocol at the study institution. Four patients were instructed to return to the clinic if symptoms continued or if complications developed, as is typical practice within our department for pediatric forearm fractures. No patients required further follow-up at a range of two to 10 years after the injury. Case II returned to the clinic at three, six, and 12 months after injury without symptoms in the wrist or evidence of growth arrest on plain radiographs. However, at the 15-month follow-up to discuss the removal of clavicle hardware, wrist radiographs demonstrated a loss of radial inclination with progressive sclerosis (Figure 4). Subsequent MRI showed a partial growth arrest with a physeal bar across the radial side of the distal radial physis (Figure 4). The patient underwent physeal bar resection and fat grafting. At 18 months post physeal bar resection, the patient had < 1 cm limb length discrepancy and no functional deficits.

**Figure 5:**
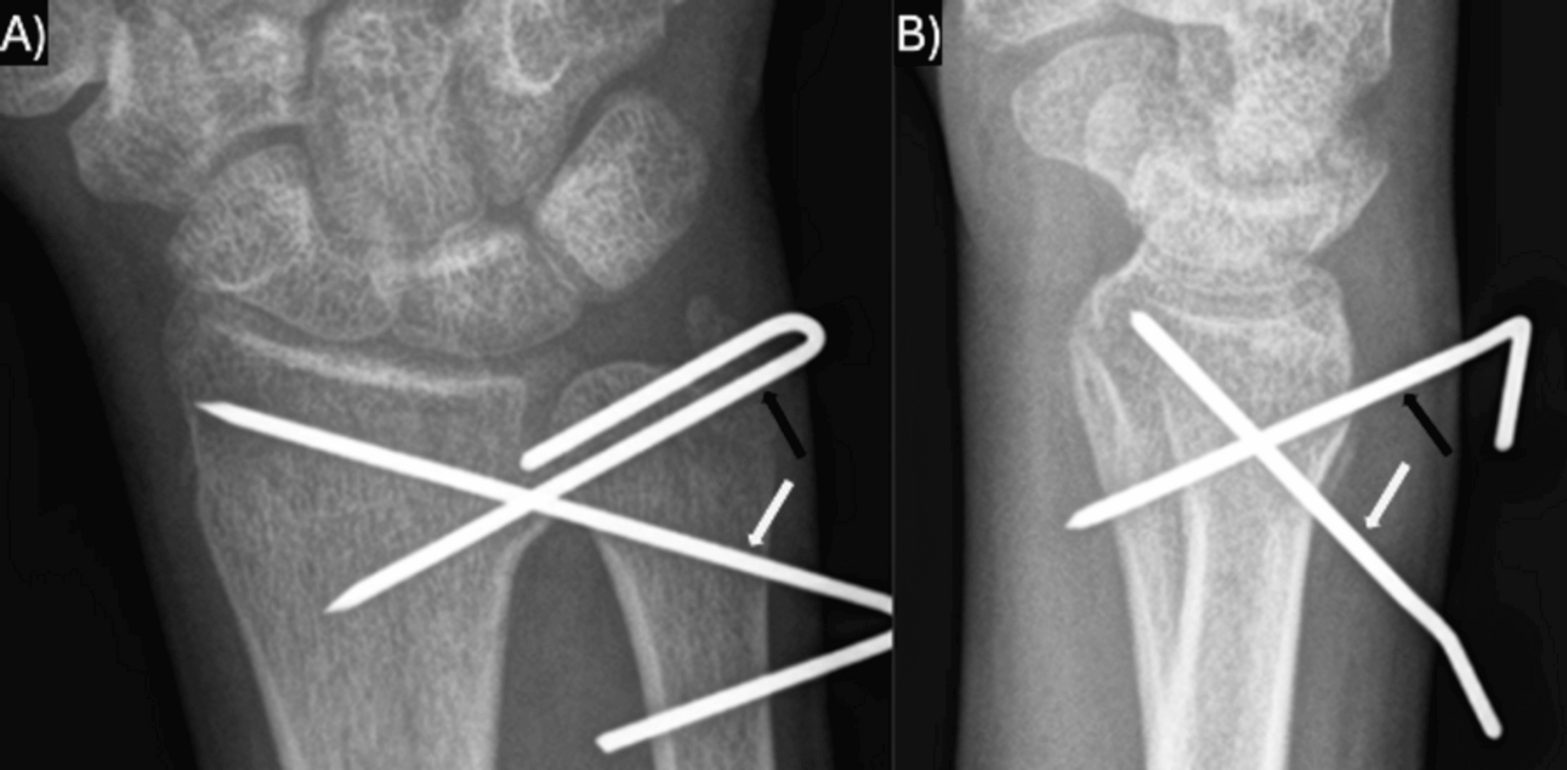
Pinning construct from after CRPP of the operatively managed case V; this case required pinning due to loss of reduction after splinting. Pins were removed at six weeks post-operatively in the clinic after the fracture was demonstrated to be healed. A) AP radiographs at six weeks postoperatively prior to pin removal; B) Lateral radiographs at six weeks postoperatively prior to pin removal. In both panels, the black arrow demonstrates the pin placed dorsally from proximal to distal, while the white arrow demonstrates the pin placed dorsally from distal to proximal. CRPP: closed reduction and percutaneous pinning; AP: anterior-posterior

One case (case V) was initially managed operatively (Figure 5). Closed reduction was attempted in the emergency department but had persistent dorsal and ulnar displacement of the ulnar lip of the distal radius. Because of the concern for DRUJ involvement and > 2 mm step-off at the physis, operative fixation was recommended by the treating surgeon to limit the possibility of altered DRUJ mechanics and physeal arrest. Surgery was performed eight days after the initial injury. Direct pressure over the fragment from the dorsal ulnar direction was used to successfully reduce the fracture under fluoroscopy in the operating room. Two 0.062 Kirschner wires (K-wires) were then inserted from the ulnar aspect of the radius, through the dorsal-ulnar fragment across the radius, to engage the far radial cortex in a crossing fashion. The reduction was confirmed on fluoroscopy, and the range of motion was evaluated in the operating room without evidence of passive blocks to motion. There was no laxity of the DRUJ with the shuck test after K-wire fixation, so the ulnar styloid fragment was not pinned. The K-wires were cut, and the forearm was placed in a sugar-tong splint with the wrist in neutral. The splint was replaced with a short arm cast ten days after surgery. At the five-week postoperative follow-up (six weeks after the injury), the cast was removed, and radiographs with the K-wires in place showed complete fracture healing (figure 5). There were no K-wire site complications, and the pins were removed at this office visit. The patient was hesitant to start the wrist range of motion after K-wire removal but had no tenderness or pain with the range of motion. The patient was instructed to return two weeks after K-wire removal for evaluation of wrist function but did not return to the clinic for follow-up.

## Discussion

Transitional fractures in adolescents most commonly occur in the distal tibia. This injury pattern is explained by the sequence of physeal closure observed in the distal tibia: central to medial to lateral [15]. Due to the predictable pattern of closure at this physis, adolescents commonly sustain either a medial SH-III fracture (Tillaux fracture) or a medial SH-III fracture on the AP radiograph and a posterior SH-II fracture on the lateral radiograph (triplane fracture). These injuries have infrequently been described in other long bones as well, including the distal radius. Prior to this case series, only 10 cases of transitional distal radius fractures had been published in the literature.

Similar to the distal tibia, the physis of the distal radius has been reported to close in a predictable fashion. Cessation of growth at the distal radius is typically complete at an average age of 17 years (range: 15 to 18 years). A sequential MRI study of 22 adolescents showed that bridging of the growth plate typically occurs from a centro-radial location, progressing ulnar to radial and finishing at the dorso-radial surface. However, in contrast to the distal tibia, physeal plate closure at the distal radius typically is complete in less than one year [14], which could contribute to the lower prevalence of transitional distal radius fractures as compared to distal tibia fractures. Additionally, transitional fractures of the distal tibia are due to torsional forces about the ankle. The distal radius is typically fractured due to axial loads and is rarely subjected to torsional forces large enough to produce a fracture [6].

The majority of reported transitional distal radius fractures, including in the present series, were due to low-energy falls, either during a sporting or recreational event or from a moderate height of less than five feet. Four cases, including case II in the present series, were due to high-energy mechanisms, one of which occurred in bilateral wrists [6-7]. All injuries were closed, and none were associated with any neurological or vascular deficits when evaluated in the emergency room or clinic.

The fracture patterns shown and described in each of the case reports are similar. The majority of cases presented with an ulnar-sided SH-III component on the AP radiographs and a dorsal SH-II component on lateral radiographs, as seen in cases I, IV, and VI above. These appear remarkably similar to the triplane fracture as described in the distal tibia. Two of the prior cases [6,8] and case II above presented with a radial rather than ulnar-sided SH-III on the AP radiograph and a complete SH-IV component on the lateral radiograph. Rarely, these cases may present with an avulsion of the ulnar styloid, but none in the present series had associated DRUJ instability on evaluation and thus did not require fixation. Cases III and V in the present series were found to have an isolated SH-III component on the AP radiograph, similar to the pattern seen in Tillaux fractures at the distal tibia; this has been reported once prior in a 16-year-old male [9].

These fractures are typically minimally displaced, although they may present with subtle radial translation [6,10], dorsal translation and angulation [7,11], or apex volar angulation [6,10,13] that may require closed reduction prior to casting. The cases in the present series were all mildly displaced with similar patterns as prior reported cases. All injuries in the present study were managed via closed reduction and splinting in the clinic or in the emergency room, with the exception of case V. This case was complicated by a dorsal ulnar-sided component oblique to the coronal and sagittal planes and involving the DRUJ that was unable to be controlled in a splint or cast. Only two of the prior reported injuries could not be controlled in a splint, one due to a severe dorsal shear pattern [6] and the other due to interposed periosteum blocking reduction [11].

Outcomes after closed management of these injuries are favorable. Seven of the 10 prior reported cases underwent closed management in a cast: six with short arm casting and one with long arm casting to prevent supination and pronation. Similarly, five of six cases in the present series were managed in a short arm cast. Treatment duration in the cast is typically six weeks after fracture occurrence with an occasional one to two more weeks of limited activity. All patients in the prior and present studies who underwent closed management returned to full activity eight weeks after the injury.

Only three patients (four fractures) from the prior literature required operative fixation for their injury. Two of the cases were due to an inability to maintain the reduction closed, while the third was due to a bilateral injury and the need for faster upper extremity functional recovery [6, 11]. Each of these cases was managed via open reduction and internal fixation (ORIF) via a modified Henry approach and volar plating of the distal radius. All four injuries healed well with no limits to activity by six or more months after surgery, and Rauer et al. reported excellent outcomes on the Quick Disabilities of the Arm, Shoulder, and Hand (DASH) score. However, both patients in the Rauer series required the removal of hardware one year after injury [6].

For fractures that are significantly displaced and require ORIF, Rauer et al. recommend a CT scan of the injury for better operative planning [6]. However, the majority of these injuries reported in the prior literature have been appropriately assessed with plain radiographs. Case II in the present study only received a CT scan of the wrist because the patient required a CT scan for other injuries. These injuries are not an absolute indication for a CT scan, and the authors of the present study recommend the utilization of advanced imaging if there is significant comminution and poor ability to properly identify the fracture fragments on plain imaging [16].

In contrast, case V is the first reported transitional fracture of the distal radius to be managed via CRPP, likely due to an inability to achieve the reduction with closed means in prior reported cases. This patient had an uneventful recovery, and the K-wires were removed six weeks after healing was confirmed on radiographs. The patient returned to full activity at that time and had not returned to the office with any issues in more than 10 years. The goal of management for transitional fractures is alignment of the articular surface, typically to less than 2 mm of displacement. The CRPP procedure is commonly used for transitional fractures of the distal tibia that can be reduced but not held by closed means [17]. Although it has had good success in the triplane fracture at the distal tibia [18-20], CRPP is more commonly used in Tillaux fractures at this location due to the simpler reduction technique [17-18]. Since case V in this series presented more similarly to a Tillaux fracture than a triplane fracture, it was likely more amenable to pinning; CRPP may, therefore, be a valid fixation method in simpler transitional wrist fractures to minimize incisions and scarring. 

Four of nine patients reported in prior studies developed findings on follow-up plain radiographs of an ulnar-sided distal radial physeal bar and increased radial angle at six or more months after injury [7-8, 11-12], and three of these four patients’ injuries were due to a high-energy mechanism. No patients had any symptoms or limited motion at that time. Case II in the present study developed a growth arrest and was found to have a radial-sided physeal bar and decreased radial inclination. The patient was asymptomatic at that time but underwent bar resection and fat grafting to try to prevent further growth disturbance. At the final follow-up, 18 months after bar resection, the patient had a < 1 cm limb length discrepancy and no functional deficits. As is the case in distal tibial transitional fractures [21-22], the minimal growth remaining at the typical age such injuries are seen likely leads to a low rate of complications from early closure of the growth plate. However, in younger patients with higher-energy injuries, such as the patient in case II of the present study, it may be of more value to monitor for physeal closure for one to two years after injury. This is an aspect of these injuries that would benefit from further study with longer clinical follow-up.

The primary limitation of this study is the small number of patients available for analysis. This is due to the inherent rarity of this injury, as evidenced by the few prior reports on transitional wrist fractures available. However, the report of these six patients is the largest consecutive series to date and adds to the growing literature on this rare injury. The second limitation is a lack of follow-up greater than the initial six- to eight-week course of treatment in five out of six patients. At our institution it is customary practice to allow pediatric patients with distal radius fractures to follow up as needed after cast removal for closed injuries, if there is no significant concern for risk of growth arrest, in order to increase convenience for patients and their families. This study demonstrates that in cases with higher energy mechanisms in younger patients, a longer follow-up period may be advised. Although this limits our follow-up and understanding of the long-term outcomes in these series, it is presumed that these five patients had no further issues with their wrists upon returning to full activity since they did not return to the clinic.

## Conclusions

To the best of our knowledge, this is the largest reported series of transitional distal radius fractures in the published literature. Additionally, these are the first two reported cases to have an isolated SH-III component on the coronal radiographs as seen in Tillaux fractures of the distal tibia. Fractures with < 2 mm physeal or articular displacement can be splinted or casted for six weeks after closed reduction if necessary. If physeal reduction cannot be maintained to within < 2 mm of displacement, the fracture should be stabilized operatively with CRPP as described in this series. Growth arrest is a risk in high-energy injuries (seen in four of five high-energy cases reported in prior literature), and patients who sustain higher-energy injuries with more than one year of growth remaining should have radiographic follow-up at regular intervals until physeal closure to assess for physeal bar formation. It is reasonable in high-energy transitional fractures to monitor more closely for growth arrest, but this complication’s impact on long-term outcomes requires further study.

## References

[1] Rosenbaum AJ, DiPreta JA, Uhl RL (2012). Review of distal tibial epiphyseal transitional fractures. Orthopedics.

[2] Peterson HA (1983). Triplane fracture of the distal humeral epiphysis. J Pediatr Orthop.

[3] Masquijo JJ, Allende V (2011). Triplane fracture of the distal femur: a case report. J Pediatr Orthop.

[4] Conroy J, Cohen A, Smith RM, Matthews S (2000). Triplane fracture of the proximal tibia. Injury.

[5] Devalentine SJ (1987). Epiphyseal injuries of the foot and ankle. Clin Podiatr Med Surg.

[6] Rauer T, Pape HC, Gamble JG, Vitale N, Halvachizadeh S, Allemann F (2020). Transitional fracture of the distal radius: a rare injury in adolescent athletes. Case series and literature review. Eur J Med Res.

[7] García-Mata S, Hidalgo-Ovejero A (2006). Triplane fracture of the distal radius. J Pediatr Orthop B.

[8] Parkar AA, Marya S, Auplish S (2014). Distal radius triplane fracture. Ann R Coll Surg Engl.

[9] Kurland A, Batko B, Hreha J, Ignatiuk A (2021). Distal radius Salter-Harris III transitional fracture in an adolescent male. Case Rep Orthop.

[10] Pearce C, Chung R (2011). Triplane fracture of the distal radius. Clin Pract.

[11] Mingo-Robinet J, Torres-Torres M, Gonzalez-Rodriguez M (2014). Triplane fracture of distal radius treated surgically: case report and review of the literature. J Pediatr Orthop B.

[12] Garcia Mata S, Hidalgo Ovejero A, Martinez Grande M (1999). Triplane fractures in the hand. Am J Orthop (Belle Mead NJ).

[13] Peterson HA (1996). Triplane fracture of the distal radius: case report. J Pediatr Orthop.

[14] Kraus R, Reyers J, Alt V, Schnettler R, Berthold LD (2011). Physiological closure of the physeal plate of the distal radius: an MRI analysis. Clin Anat.

[15] Olgun ZD, Maestre S (2018). Management of pediatric ankle fractures. Curr Rev Musculoskelet Med.

[16] Little JT, Klionsky NB, Chaturvedi A, Soral A, Chaturvedi A (2014). Pediatric distal forearm and wrist injury: an imaging review. Radiographics.

[17] Wuerz TH, Gurd DP (2013). Pediatric physeal ankle fracture. J Am Acad Orthop Surg.

[18] Lintecum N, Blasier RD (1996). Direct reduction with indirect fixation of distal tibial physeal fractures: a report of a technique. J Pediatr Orthop.

[19] Çiçekli Ö, Özdemir G, Uysal M, Biçici V, Bingöl İ (2016). Percutaneous cannulated screw fixation for pediatric epiphyseal ankle fractures. Springerplus.

[20] Castellani C, Riedl G, Eberl R, Grechenig S, Weinberg AM (2009). Transitional fractures of the distal tibia: a minimal access approach for osteosynthesis. J Trauma.

[21] Ertl JP, Barrack RL, Alexander AH, VanBuecken K (1988). Triplane fracture of the distal tibial epiphysis. Long-term follow-up. J Bone Joint Surg Am.

[22] Barmada A, Gaynor T, Mubarak SJ (2003). Premature physeal closure following distal tibia physeal fractures: a new radiographic predictor. J Pediatr Orthop.

